# Cadaver Models in Residency Training for Uncommonly Encountered Ultrasound-Guided Procedures

**DOI:** 10.1177/2382120519885638

**Published:** 2019-11-19

**Authors:** Richard Amini, Luis D Camacho, Josephine Valenzuela, Jeannie K Ringleberg, Asad E Patanwala, Jack Stearns, Elaine H Situ-LaCasse, Josie Acuña, Srikar Adhikari

**Affiliations:** 1 Department of Emergency Medicine, The University of Arizona, Tucson, AZ, USA; 2 College of Medicine, The University of Arizona, Tucson, AZ, USA; 3 Sydney Pharmacy School, The University of Sydney, Sydney, NSW, Australia; 4 Department of Molecular and Cellular Biology, The University of Arizona, Tucson, AZ, USA

**Keywords:** Point of care ultrasound, bedside ultrasound, ultrasound education, emergency ultrasound, ultrasound guidance, thoracentesis, regional nerve block, nerve block, cadaver, internship and residency

## Abstract

**Background:**

Arthrocentesis of the ankle and elbow and brachial plexus nerve blocks are infrequently performed procedures; however, clinicians in specialties such as emergency medicine are required to be proficient in these procedures in the event of emergent or urgent necessity.

**Objectives:**

The objective of this study was to create, implement, and assess a fresh cadaver-based educational model to help resident physicians learn how to perform ultrasound-guided arthrocentesis of the ankle and elbow and ultrasound-guided regional nerve blocks.

**Methods:**

This was a single-center cross-sectional study conducted at an academic medical center. After a brief didactic session, 26 emergency medicine residents with varying levels of clinical and ultrasound experience rotated through 4 fresh cadaver-based stations. The objective of each station was to understand the sonographic anatomy and to perform ultrasound-guided arthrocentesis or regional nerve block with hands-on feedback from ultrasound fellows and faculty. Participants were subsequently asked to complete a questionnaire which evaluated participants’ experience level, opinions, and procedural confidence regarding the 4 stations.

**Results:**

A total of 26 residents participated in this study. All 26 residents agreed that the cadaver model (compared with clinical anatomy) was realistic regarding ultrasound quality of the joint space, ultrasound quality of the joint effusion, ultrasound quality of nerves, tissue density, needle guidance, and artifacts. Finally, there was a statistically significant difference between mean scores for pre-simulation and post-simulation session participant procedural confidence for all 4 procedures.

**Conclusions:**

This fresh cadaver-based ultrasound-guided educational model was an engaging and well-received opportunity for residents to gain proficiency and statistically significant confidence in procedures which are uncommonly performed in clinical settings.

## Introduction

Arthrocentesis of the ankle and elbow and regional brachial plexus blocks are infrequently performed procedures; however, clinicians in specialties such as emergency medicine (EM) are required to be proficient in these procedures in the event of emergent or urgent necessity.^1^ The use of ultrasound (US) has been shown to improve success and decrease procedural complication risk,^2,3^ which may be further diminished by improving procedural familiarity and competence through simulation education. During residency training, it is important that novice clinicians be exposed to various opportunities to develop skills through deliberate practice.^4^ The need for training supplementation had led to various innovations in procedural training through the use of simulations, high-fidelity US models, and fresh cadaver models.^5-15^ Simulation sessions, such as those found in mannequin labs or fresh cadaver labs, can provide the precise learning environment for deliberate practice of both common and uncommonly performed procedures.^1,13-18^

Simulation-based training allows residents to learn technical skills in a safe, low-stress environment while protecting patients from risks associated with invasive procedures in the hands of novices. Although simulation-based instruction can provide high-quality training, there are substantial differences in the tactile and anatomic features of the human body when compared with the manikin and phantom models that are commonly used. In recent years, utility of fresh cadaver models for training in central line placement, thoracentesis, and thoracostomy have been demonstrated to be well received.^9,17^ The objective of this study was to create, implement, and assess a fresh cadaver-based educational model to help resident physicians learn how to perform US-guided arthrocentesis of the ankle and elbow and US-guided regional nerve blocks.

## Methods

### Setting and population

This was a single-center cross-sectional study conducted at an academic medical center. The section of Emergency Ultrasound within the Department of Emergency Medicine has an Institution-Review-Board-approved umbrella study for education-related US studies. This study was internally reviewed prior to inclusion in the approved study. Our institution teaches residents from 3 different programs (Banner University Medical Center—Tucson Campus Emergency Medicine Residency, Banner University Medical Center—Tucson Campus Emergency Medicine/Pediatrics Combined Residency, and Banner University Medical Center—South Campus Emergency Medicine Residency). In total, there are 79 residents. In this study, 26 (33%) of the 79 residents participated. Participation in this study was voluntary, and participants provided consent prior to participation. Data were gathered in February 2019. All bodies used in our fresh cadaver lab were provided by the College of Medicine Willed Body Program and selflessly donated by our donors and their families.

### Study design

As part of the resident training curriculum at our institution, residents have periodic breaks from didactic lectures for the purpose of hands-on training sessions. A 1-day educational session focused on point-of-care US for uncommonly performed procedures was integrated into one of these sessions. Instructors for this course were 3 Department of Emergency Medicine faculty and attending physicians with US fellowship training.

#### Educational curriculum

At the start of the session, a 30-minute didactic lecture was delivered to participating residents to review US anatomy, sonographic appearance of joint effusions, and procedural techniques involved in US-guided arthrocentesis of the ankle and elbow as well as regional anesthesia of the brachial plexus and forearm nerves. During this presentation, clinical cases were used to express the necessity for emergency physicians to be competent in arthrocentesis and regional nerve blocks. Furthermore, these clinical cases were used to demonstrate how US guidance can help clinicians perform these infrequently encountered procedures with significant ease.

**Figure 1.**
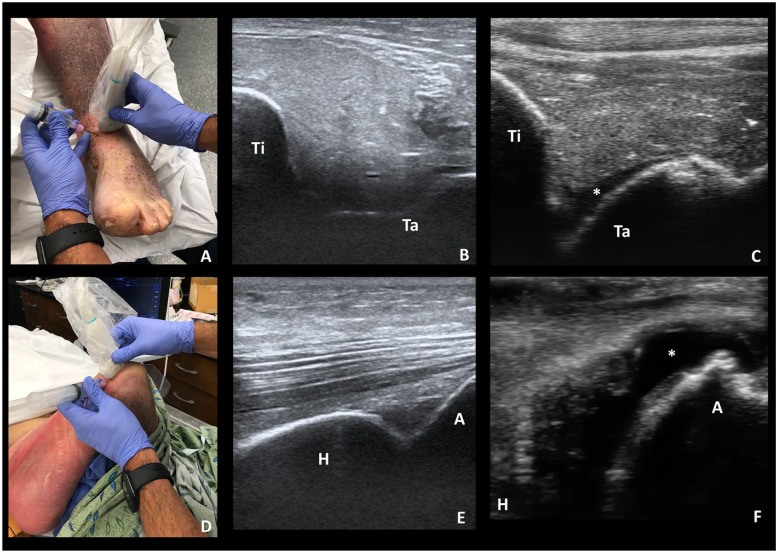
(A) Fresh frozen cadaver ankle model; (B) ankle imaging of the tibia (Ti), talus (Ta), and joint space; (C) ankle imaging of the tibia (Ti), talus (Ta), and an ankle joint effusion indicated with an asterisk; (D) fresh frozen cadaver elbow model; (E) elbow imaging of the humerus (H), acromion (A), and notable triceps tendon; (F) elbow imaging of the humerus (H), acromion (A), and an elbow joint effusion indicated with an asterisk.

**Figure 2.**
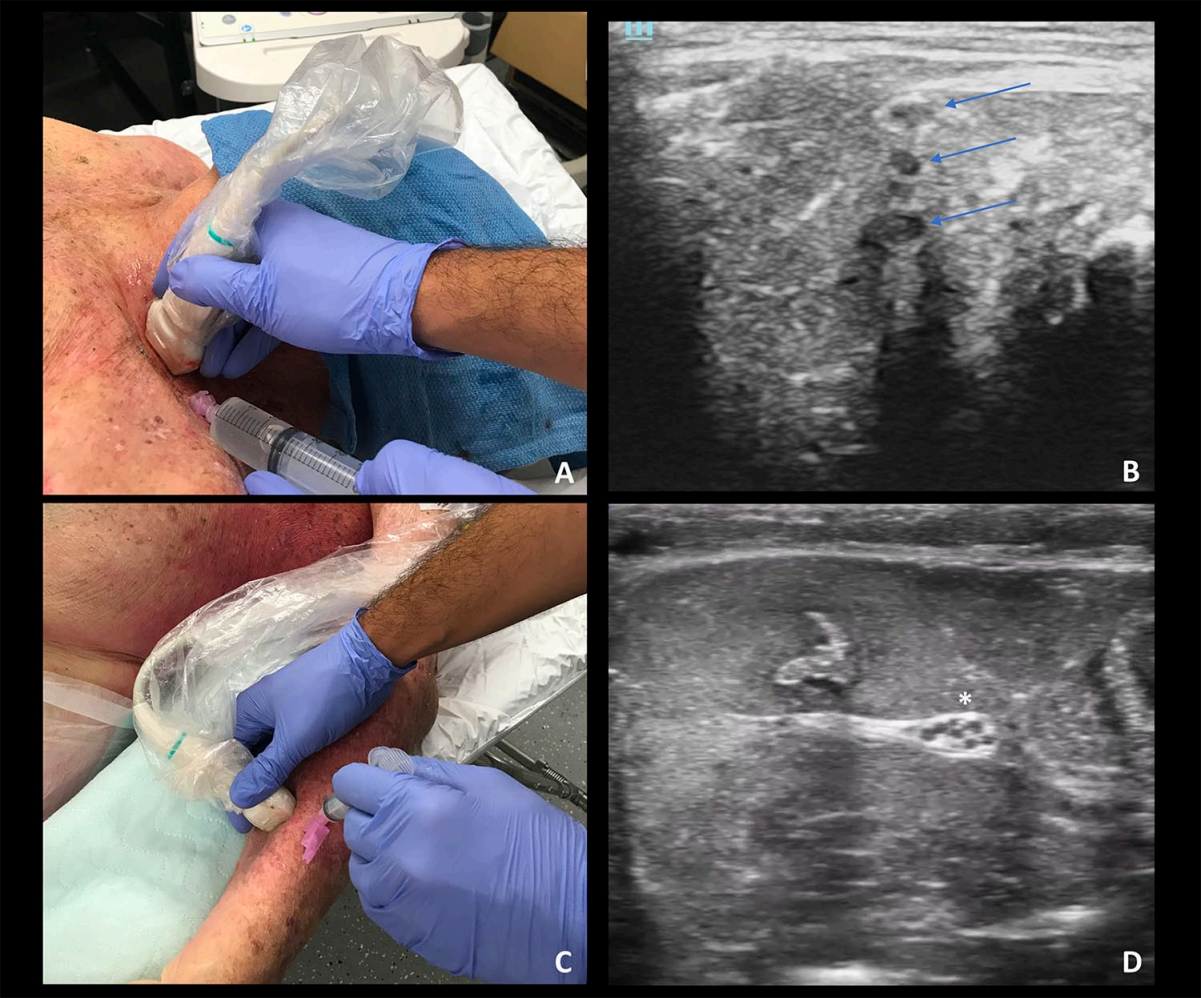
(A) Fresh cadaver brachial plexus model; (B) interscalene imaging of cervical nerves: C4—upper arrow, C5—middle arrow, and C6—lower arrow; (C) fresh cadaver forearm nerve model; (D) forearm imaging, with asterisk indicating the median nerve.

Unlike embalmed cadavers which have been perfused with preservatives and air, fresh frozen cadavers maintain excellent sonographic quality and are ideal for US educational models. The echogenicity of bones, tendons, and injected fluid are undifferentiable from clinical images (Figures 1 and 2). Furthermore, fresh frozen cadavers can be manipulated easily for procedural education. For our educational session, the cadavers were manipulated into positioning similar to clinical settings and conducive to arthrocentesis and regional anesthesia blocks. For arthrocentesis, the ankle was dorsiflexed to 90 degrees and the leg placed supine (Figure 1A); the elbow was flexed to 90 degrees and the shoulder internally rotated (Figure 1D). These positions place the US machine in direct line of site to the operator, thus providing a more ergonomic procedure. Similarly, for the brachial plexus, the cadaver was supine and the neck was rotated to the contralateral side increasing access to the interscalene muscles and brachial plexus (Figure 2A); for the forearm nerve block, the cadaver and arm were supine and the wrist was supinated providing access to the volar aspect of the mid-forearm (Figure 2C). This positioning optimized clinician comfort while performing the procedure with the US machine in line of sight.

Resident physicians rotated through 4 unique proctored training stations: US-guided ankle arthrocentesis, US-guided elbow arthrocentesis, US-guided interscalene nerve block, and US-guided forearm nerve blocks (Table 1). In each station, the participant was proctored through the sonographic assessment of the joint space or anatomical region. Subsequently, the participant was provided feedback as they used US to guide an 18-gauge needle into the joint space or toward the identified nerves (Figures 1 and 2).

**Table 1.** Procedural station description.

Procedure station description	
Ultrasound-guided procedure station	Learning objectives
Ankle effusion/arthrocentesis	*How to evaluate the ankle for effusion and conduct an ankle arthrocentesis.* Review the sonographic evaluation of the tibiotalar joint in the long axis and axes for the presence of a joint effusion. Practice ultrasound-guided arthrocentesis of the tibiotalar joint. Each participant practices a minimum of 1 ultrasound examination and 1 arthrocentesis.
Elbow effusion/arthrocentesis	*How to evaluate the elbow for effusion and conduct an elbow arthrocentesis.* Review the sonographic evaluation of the humeroulnar joint in the long and short axes for the presence of a joint effusion. Practice ultrasound-guided arthrocentesis of the humeroulnar joint. Each participant practices a minimum of 1 ultrasound examination and 1 arthrocentesis.
Interscalene regional nerve block	*How to identify and perform a regional nerve block of the brachial plexus.* Review the sonographic anatomy of the brachial plexus between scalene muscles of the neck. Practice ultrasound-guided regional nerve block of the brachial plexus. Each participant evaluates the anatomy and performs minimum 1 regional nerve block
Forearm regional nerve block	*How to identify and perform a regional nerve block of the forearm nerves.* Review the sonographic anatomy of the forearm. Practice ultrasound-guided regional nerve block of the ulnar and median nerves. Each participant evaluates the anatomy and performs a minimum of 1 regional nerve block.

### Assessment

The participants were asked to fill out a questionnaire at the end of the training session. The questionnaire included items regarding previous exposure to US-guided procedures, opinions regarding the educational intervention, and confidence in US skills using a 4-point unanchored Likert-type scale (0-3).

### Data analysis

All analyses were conducted in Stata 11 (StataCorp LP, College Station, TX). Data are presented as means and percentages with 95% confidence intervals. A paired *t* test was used for comparison of paired samples. The statistical level of significance was set at *P* < .05.

## Results

A breakdown of the residents by postgraduate year (PGY) of training and average number of completed procedures is summarized in Table 2. A total of 26 out of 79 residents participated in this study. All 26 residents completed the questionnaire before and after the educational session. All 26 residents agreed that the cadaver model (compared with clinical anatomy) was realistic regarding US quality of the joint space, US quality of the joint effusion, US quality of nerves, tissue density, needle guidance, and artifacts. Finally, there was a statistically significant difference between mean scores for pre-simulation and post-simulation session participant procedural confidence for all 4 procedures (Table 3).

**Table 2.** PGY breakdown and procedural confidence.

PGY class (n)	Number of elbow or ankle arthrocentesis performed	Number of brachial plexus blocks performed	Number of forearm blocks performed
PGY-1 (9)	100% had performed ⩽ 3	100% had performed ⩽ 3	100% had performed ⩽ 3
PGY-2 (10)	100% had performed ⩽ 3	100% had performed ⩽ 3	100% had performed ⩽ 3
PGY-3 (7)	86% had performed ⩽ 3	100% had performed ⩽ 3	100% had performed ⩽ 3

Abbreviation: PGY, postgraduate year.

**Table 3.** Procedural confidence (Likert-type scale, 0-3).

Item	Pre-simulation mean (95% CI)	Post-simulation mean (95% CI)	*P* value
How confident were you in the sonographic diagnosis of joint effusions of the ankle and elbow?	1.4 (1.1-1.7)	2.3 (2.1-2.5)	<.001
How confident were you in ultrasound-guided arthrocentesis of the ankle and elbow?	1.2 (0.9-1.5)	2.3 (2.1-2.5)	<.001
How confident were you in ultrasound-guided nerve blocks of the forearm?	1.0 (0.7-1.2)	2.0 (1.8-2.2)	<.001
How confident were you in ultrasound-guided nerve blocks of the neck (supraclavicular and interscalene)?	0.2 (0.1-0.4)	1.5 (1.2-1.8)	<.001

Abbreviation: CI, confidence interval.

## Discussion

Our study results indicate that fresh frozen cadavers can successfully be used for the education and practice of arthrocentesis and regional anesthesia. To our knowledge, this study is the first to use fresh frozen cadavers for residency training in US-guided regional anesthesia of the brachial plexus. Our study participants unanimously agreed that US imaging of the fresh frozen cadaver was realistic compared with images acquired in the clinical setting. Furthermore, our educational model was useful for teaching US-guided procedures to novice residents. Our study demonstrated a statistically significant improvement in operator confidence for all 4 procedures. Our results strengthen the evidence that fresh cadaver models for US-guided procedures are useful models for teaching procedures which are infrequently encountered.

In our fresh cadaver arthrocentesis model, we trained the residents on how to first use US to identify the presence or absence of joint effusions. Subsequently, residents were encouraged to perform arthrocentesis, thereby improving the operators’ diagnostic and procedural skills. These skills are necessary because elbow and ankle joint effusions are less frequently encountered. As Adhikari et al demonstrated in 2010, only 38% of patients with elbow pain and swelling were found to have elbow joint effusions and only 15% of patients with ankle pain and swelling were found to have ankle joint effusions. Furthermore, 65% of the time, physician management plans were altered when US was used to evaluate the joint space prior to arthrocentesis.^19^ This literature demonstrated the necessity for clinicians to be proficient with US so that they can diagnose joint effusions accurately and subsequently perform arthrocentesis. A recent study by Berona et al evaluated the success rates between landmark and US-guided arthrocentesis.^20^ Although their data were inconclusive, Adhikari et al’s^19^ study demonstrated the need for clinicians to be confident in the US diagnosis of joint effusion as this can change management. Our arthrocentesis stations were successfully created with fresh frozen cadavers to educate the diagnosis of joint effusion as well as the arthrocentesis. It was well received by resident physicians, and it was easily integrated into the Emergency Medicine residency program curriculum.

Similarly, our regional anesthesia stations were successfully created with fresh frozen cadavers, and these stations provided the residents to learn sonographic anatomy and procedural competency of brachial plexus and forearm nerve blocks. We chose to teach these 2 regional blocks because they vary in both difficulty and familiarity. A recent study demonstrated that brachial plexus or interscalene nerve blocks are performed at only 33% of academic EM centers, whereas forearm nerve blocks are performed at 74% of academic EM centers.^21^ Our cadaver model can provide residency physicians additional training opportunities for regional anesthesia in controlled settings where purposeful practice can take place. Images obtained using the fresh frozen cadavers were comparable to those obtained on patients and the tissue density and echogenicity was excellent for training.

This study is not without limitations. Our sample size of 26 is small which limits the conclusions reached. We used a convenience sample of residents who were present during conference, which may have introduced selection bias. Furthermore, we did not assess for procedural competency but rather evaluated residence confidence in the procedures, which limits our ability to demonstrate this training session as an effective training module. Finally, access to cadavers is limited and our institution is privileged to be a partner of the Willed Body Program that allows us a steady supply of cadavers. Additional research should seek to assess knowledge acquisition and long-term retention.

## Conclusions

This fresh cadaver-based US-guided educational model was an engaging and well-received opportunity for residents to gain proficiency and statistically significant confidence in procedures which are uncommonly encountered in clinical settings.
